# Elevated Fatty Acid Binding Protein 4 (FABP4) Associated With Liver Damage and Kidney Complications in Thalassemia Patients

**DOI:** 10.1155/bmri/3090639

**Published:** 2026-03-23

**Authors:** Rawia Yusef Al Belbesi, Manal Mohammed Abbas, Ali Abu Siyam, Sima Zein

**Affiliations:** ^1^ Department of Medical Laboratory Sciences, Faculty of Allied Medical Sciences, Al-Ahliyya Amman University, Amman, Jordan, ammanu.edu.jo; ^2^ Department of Medical Laboratory Sciences, Faculty of Allied Medical Sciences, Jadara University, Irbid, Jordan, jadara.edu.jo; ^3^ Department of Pharmaceutical Biotechnology, Faculty of Allied Medical Sciences, Al-Ahliyya Amman University, Amman, Jordan, ammanu.edu.jo

**Keywords:** adipokines, chronic diseases, FABP4, thalassemia major

## Abstract

**Background:**

Thalassemia is considered a significant health concern. Previous studies had shown that numerous adipokines, including fatty acid binding protein 4 (FABP4), might contribute to the development of complications in thalassemia.

**Objectives:**

This study is aimed at investigating FABP4 levels in thalassemia major patients receiving regular blood transfusions as well as to explore the association between FABP4 and target organ functioning as kidney and liver.

**Method:**

Serum samples were collected from 154 individuals with thalassemia major and subjected to various analyses, including hematological assessments, biochemical evaluations (kidney and liver functions), and immunological screenings using enzyme‐linked immunosorbent assay (ELISA).

**Results:**

Examination of sociodemographic and disease‐related factors indicated osteoporosis emerging as the predominant comorbidity. Elevated ferritin levels, an increased platelets (PLT) count, higher nucleated red blood cell (NRBC) count, and elevated bilirubin (BIL) levels in patient serum were observed. Conversely, creatinine levels were significantly low, with a mean of 21.7 and 25.4 *μ*mol/L in females and males, respectively. FABP4 levels exhibited more frequent increases in females of *p* value = 0.03, particularly in the age groups of (13–18, 19–30) years; respectively, FABP4 levels had a significant difference of *p* value = 0.003 with the aspartate aminotransferase/alanine aminotransferase (AST/ALT) ratio.

**Conclusion:**

A significant difference was seen between serum FABP4 and bilirubin and AST/ALT ratio, showing a connection between serum FABP4 and liver injury and kidney complications. Consequently, while further studies are essential for a comprehensive exploration of this hypothesis, serum FABP4 may have a potential role in chronic diseases in thalassemia patients.

## 1. Introduction

Thalassemia is one of the most prevalent genetic disorders worldwide [1, 2], with an estimated carrier prevalence of approximately 2%–4% in Jordan [3, 4]. It is an inherited hematological disorder characterized by chronic hemolytic anemia resulting from defective hemoglobin (Hb) synthesis, which shortens the lifespan of red blood cells (RBCs). The condition arises from genetic mutations that disrupt the production of *α*‐ or *β*‐globin chains, giving rise to *α*‐ and *β*‐thalassemia, respectively [5, 6]. The most severe form, *β*‐thalassemia major (*β*TM), occurs when both *β*‐globin genes are defective, leading to anemia that requires lifelong medical management [7, 8, 9]. Patients with *β*TM typically depend on regular blood transfusions, which often result in iron overload and oxidative stress that predominantly affect the liver and can progress to cirrhosis if not detected early [10–13]. Iron accumulation also contributes with cardiomyopathy, endocrine dysfunction, renal impairment, and chronic inflammation. The generation of reactive oxygen species (ROS) and lipid peroxidation exacerbate cellular damage, thereby increasing the risk of heart failure, hepatic fibrosis, and other metabolic complications [14–20]. Although iron is essential for normal cellular function, excess iron promotes free radical formation that damages carbohydrates, proteins, lipids, and nucleic acids [21].

Fatty acid binding proteins (FABP4) are a family of small cytoplasmic proteins that regulate intracellular lipid transport and metabolism, exhibiting a high affinity for both saturated and unsaturated fatty acids [22, 23]. Among them, FABP4, also known as adipocyte FABP, is an adipokine that plays a key role in metabolic and inflammatory pathways. Elevated circulating FABP4 levels have been associated with atherosclerosis, coronary artery disease, and several metabolic disorders, including nonalcoholic steatohepatitis, dyslipidemia, and heart failure [24–26]. Emerging evidence suggests that adipokines such as FABP4 may contribute to the pathophysiology of thalassemia‐related complications by promoting lipid accumulation and inflammation in nonadipose tissues [27–29]. Therefore, this study is aimed at assessing serum FABP4 levels in patients with *β*TM and determining their relationship with iron overload, inflammation, and target organ function, particularly in the liver and kidneys. The finding highlights the potential of FABP4 as a novel biomarker for predicting metabolic complications in thalassemia.

## 2. Materials and Methods

### 2.1. Study Population

A control case study was performed on 154 Jordanian volunteers in the north Jordan. Patients who had prediagnosed with TM and were receiving regular blood transfusion and chelation therapy were included. Non‐Jordanian Individuals and Jordanians who had not received blood transfusion therapy were excluded in the study. Samples were collected at Princes Rahma Governmental Hospital, Irbid, Jordan. The patient group had a mean age of 20.2 years, whereas the control group compromised 51 healthy individuals with a mean age of 21.2 years.

### 2.2. Participants Information, Data Collection, and Blood Sampling

Written consent was taken from all volunteered individuals per the instructions of the ethical committee of Jordan MOH (Approval NO.MBA/12643/1‐9‐2021). Venous blood samples, 3–5 mL, from each participant after overnight fasting, blood samples were collected into EDTA and plain tube.

### 2.3. Hematological Investigations: Complete Blood Count (CBC) and Ferritin

The CBC was performed for 154 patients′ samples by a Sysmex XP‐300 hematology analyzer. Serum ferritin level was measured by Cobas e 411 immunoassay analyzer.

### 2.4. Biochemical Investigations: Kidney and Liver Functions

Kidney and liver function parameters were measured using commercial kits (Roche Diagnostic, United States) on the Cobas c 411 analyser.

### 2.5. Immunological Investigation

The serum concentration of FABP4 was assessed using a commercially available sandwich ELISA (Minneapolis, United States). The assay was performed following the manufacturer procedure as the following, a calibration curve was generated by plotting absorbance values at 450 nm against FABP4 concentrations of the calibrators, and unknown sample concentrations were determined using this curve.

## 3. Results

### 3.1. Patients Sociodemographic Characteristics

The characteristics of 154 beta‐thalassemia patients in this study were listed in Table 1, including the mean age of patients, age range, and gender. Almost half of the patients did not have siblings with beta‐thalassemia, but nearly a third had one sibling with this disorder.

**Table 1:** Descriptive statistics of the characteristics of the patient sample.

**Numerical variable**	**M** **e** **a** **n** ± **S** **D**	**Median**	**Range**	**Missing (%)**

Age	20.2 ± 9	19	3–42	3 (1.9%)
Number of transfused blood units in the last session	1.5 ± 0.6	2	1–5	
Monthly average number of transfused blood units	1.9 ± 0.5	2	1–5	

**Categorical variable**	**Number**		**Percentage**	

Gender				
Male	87		56.5%	
Female	67		43.5%	
Smoking status				
Smoker	51		33.1%	
Nonsmoker	103		66.9%	
Number of siblings with beta‐thalassemia				
0	71		46.1%	
1	51		33.1%	
2	25		16.2%	
3‐5	7		4.4%	
Iron chelating agent used				
Ferriprox (deferiprone)	22		14.3%	
Exjade (deferasirox)	120		77.9%	
Desferal	27		17.5%	
I do not know	3		1.9%	
Missing	1		0.6%	
Number of chelating agents used	133		86.4%	
1	15		9.7%	
2	2		1.3%	
3	3		1.9%	
I do not know	1		0.6%	
Splenectomy	59		38.3%	
Chronic diseases				
Allergies	2		1.3%	
Type I diabetes	1		0.6%	
Type II diabetes	11		7.1%	
Thyroid dysfunction	5		3.2%	
Thrombosis	2		1.3%	
CNS and neurological	2		1.3%	
Cardiac disease	2		1.3%	
Osteoporosis disease	17		11%	
Chronic kidney disease	1		0.6%	
Hepatobiliary disease	7		4.5%	
Gastrointestinal disease	1		0.6%	
Other blood disorders	6		3.9%	
Cancer	1		0.6%	
I do not know/have	114		74%	
Regularly taken mineral and vitamin supplements				
Folic acid				
Vitamin‐C	152		95%	
Vitamin‐D	23		14.37%	
Vitamin‐B12	118		73.75%	
Zinc supplement	23		14.37%	
Calcium supplement	18		11.25%	
	96		60%	

### 3.2. Disease Related Characteristics

Some of the thalassemia disease‐related characteristics as received treatments, blood transfusion experience, and comorbidity were listed in Table 1. Patients were asked to indicate their regularly taken iron chelating therapy, the majority of them were found to take Exjade; however, the patients received approximately two blood units monthly. Two third of thalassemia patients had undergone splenectomy. The majority of them were taking folic acid to reduce the severity of thalassemia complications that are mentioned in Table 1 among other supplements such as vitamin D, B12, and calcium.

### 3.3. Hematological and Biochemical Investigation Results

The laboratory test findings of the study sample are shown in Table 2, where the means of kidney function tests as sodium (Na), potassium (K), calcium (Ca), and urea were within normal range, whereas creatinine was low in both females and males, respectively. On the other hand, the mean of liver function tests total bilirubin (TBIL) and direct bilirubin (DBIL), alkaline phosphatase (ALP), aspartate aminotransferase (AST), and alanine aminotransferase (ALT) were higher than normal. Furthermore, mean ferritin showed significantly high levels. According to CBC results, the means of white blood cells (WBC) count were slightly higher than normal. The number of nucleotide red blood cells (NRBCs) was high. Moreover, blood platelets (PLTs) counts were high, whereas the mean Hb level was below normal in thalassemia patients. In addition, the mean corpuscular volume (MCV) was slightly lower than normal range, whereas the mean corpuscular hemoglobin (MCH) and mean corpuscular hemoglobin concentration (MCHC) were within normal range. The RBCs count and hematocrit (HCT) were also lower than normal levels in the thalassemia patients.

**Table 2:** The hematological and biochemical laboratory tests of thalassemia patients.

Laboratory tests^a^	Normal range	*M* *e* *a* *n* ± *S* *D*
Kidney Function Test		
Na: mmol/L	135.00–145.00	137.06 ± 2.38
K: mmol/L	3.50–5.20	4.49 ± 0.39
Ca; mmol/L	2.10–2.60	2.25 ± 0.17
Urea: mmol/L	2.10–8.50	4.69 ± 1.33
Creatinine; *μ*mol/L	59.00–104.00 M	25.4 ± 12.85
	45.00–84.00 F	21.78 ± 9.91
Liver Function Test		
TBIL; mg/dl	0.10–1.20	2.15 ± 1.34
DBIL; mg/dl	0.00–0.300	0.52 ± 0.18
ALP; U/L	44.00–147.00	173.49 ± 105.53
AST; U/L	10.00‐40.00 M	43.61 ± 28.53
	9.00–32.00 F	35.7 ± 32.62
ALT; U/L	7.00–40.00 M	39.81 ± 34.6
	7.00–35.00 F	34.46 ± 28.02
Ferritin Level; ng/ml	24–336 M	3116 ± 2552
	25–200 F	
Blood Cell Count Test		
WBC Counts; 109/L	4.50–11.00	11.3 ± 6.6
RBC Counts; 1012/L	4.70–6.10 M	3.0 ± 0.4
	4.20–5.40 F	
Hb level; g/dL	13.20–16.60 M	7.9 ± 0.90
	11.60–15.00 F	
HCT; %	41.00–50.00 M	
	36.00–48.00 F	24.2 ± 3.2
MCV; fL	80.00–100.00	81 ± 5.4
MCHC level; g/dL	32.00–36.00	32.8 ± 1.6
MCH level; pg	27.50–33.20	26.5 ± 1.7
Blood PLTs Count; ×10^3^/*μ*L	150.00–450.00	499 ± 302
NRBC/100 WBC	0.00	49.03 ± 109

Abbreviations: ALP, alkaline phosphatase; ALT, alanine aminotransferase; AST, aspartate transaminase; Ca, calcium; DBIL, direct bilirubin; F, female; Hb, hemoglobin; HCT, hematocrit; K, potassium; M, male; MCH, mean corpuscular Hb; MCHC, mean corpuscular Hb concentration; MCV, mean corpuscular; Na, sodium; NRBC, number of nucleated RBCs; PLTs, platelets; RBC, red blood cell; TBIL, total bilirubin; WBC, white blood cell.

### 3.4. Comparative Outcomes Stratified by FABP4 Median

**Figure 1:**
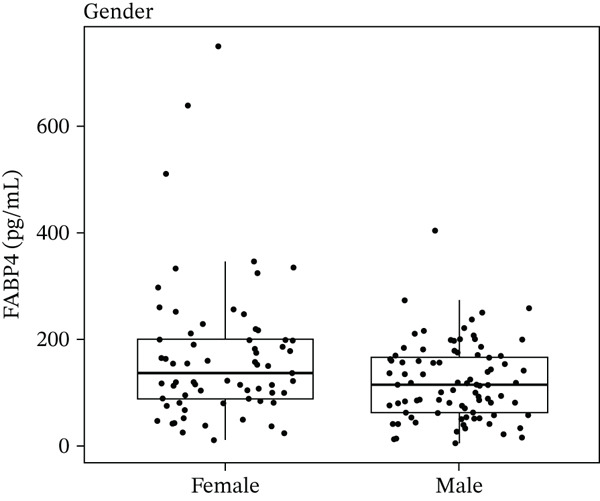
Boxplot of FABP4 levels in gender groups.

There was a significant difference in FABP4 between males and females as shown in Figure 1, where FABP4 median value was 136 pg/mL in females compared with 114 pg/mL in males with significant value of (*p* = 0.031).

Moreover, FABP4 varies according to age, where age group of (13–18) years and (19–30) years had higher FABP4, but these differences in FABP4 between age categories were not significant as Table 3 indicated.

**Table 3:** Categories of age in patients and the corresponding FABP4 levels.

Variable	*n* (%)	FABP4		*P* ^∗^	Rho, *P* ^∗∗^
		*M* *e* *a* *n* ± *S* *d*	Median (IQR)		
Age					
Group	32 (20.8%)	129 ± 75	126 (75–198)		
3–12	43 (27.9%)	138 ± 80	124 (79–180)		
13–18	52 (33.8%)	157 ± 145	117 (79–163)	0.982	0.01, 0.891
19–30	24 (15.6%)	127 ± 62	116 (83–176)		
31–42	3 (2.0%)	97 ± 115	51 (32–140)		
missing					

Abbreviations: IQR, interquartile range; Sd, standard deviation.

^∗^P value is from Kruskal–Wallis omnibus test.

^∗∗^Spearman′s rank correlation rho and p value.

In addition, there was no significant correlation between FABP4 and age (rho = 0.010, *p* = 0.891).

### 3.5. Relationship Between Liver Function and FABP4

The mean ferritin for the patients was 3116 (±2552), with a median value of 2104. The minimum ferritin value was 253 and the maximum value was 12269. No significant correlation was found between patient ferritin and FABP4 (*r* = −0.016, *p* value = 0.846) (Table 4).

**Table 4:** Categories of ferritin level based on median in male and female patients and the corresponding FABP4 levels. ∗ *P* value is from Mann–Whitney *U* test. IQR: Interquartile range. sd: standard deviation.∗∗Spearman′s rank correlation rho and*p*value.

Ferritin ng/mL [*N* = 154]	*n* (%)	FABP4		*P*∗	Rho, *P*∗∗
		*M* *e* *a* *n* ± *s* *d*	Median (IQR)		
≤ Median	78 (50.6%)	133 ± 79.2	119 (71.8–188)	0.881	−0.02, 0.846
> Median	76 (49.4%)	147 ± 125	117 (79.6–175)		

Median ferritin levels in males and females (2773 and 1955 ng/mL, respectively) were used to divide the patient group into a group at or below the median and a group above the median). Median FABP4 values in both groups were similar (*p* = 0.881) (Table 4).

Patient ALT was grouped based on the normal range. The mean of FABP4 was the highest among patients group with high ALT (Table 5). These differences in FABP4 were not significant after *p* value adjustment (*p* = 0.114). Moreover, ALT was marginally significantly correlated with FABP4 before *p* value adjustment (*r* *h* *o* = 0.16, *p* = 0.050, adj. *p* = 0.100).

**Table 5:** Categories of liver function indicators in patients and the corresponding FABP4 levels.

Liver function indicators	*n* (%)	FABP4		*P* ^∗^	Adj. *P* ^∗∗^	Rho, *P* value ^∗∗∗^
		*M* *e* *a* *n* ± *S* *d*	Median (IQR)			
ALT [*N* = 149]						
Low (< 7 U/L)	10 (7%)	115 ± 72	101 (72–153)			
Normal (M:7–40, F:7–35 U/L)	82 (57.3%)	131 ± 100	114 (63–174)	0.038	0.114	0.16, 0.050
High (M: > 40, F: > 35 U/L)	51 (35.7%)	166 ± 118	156 (86–198)			
AST [*N* = 149]						
Normal (M: 10–40, F: 9–32 U/L)	89 (62.2%)	133 ± 98	118 (79–177)	0.121	0.145	0.01, 0.906
High (M: > 40, F: > 32 U/L)	54 (37.8%)	158 ± 118	147 (81–194)			
AST/ALT [*N* = 149]						
Low (< 1)	42 (29.4%)	174 ± 115	156 (97–209)	0.005	0.030	−0.26, 0.001
Normal (1–2)	86 (47.6%)	147 ± 110	136 (86–191)			
High (> 2)	33 (23.1%)	93 ± 63	83 (46–124)			
ALP [*N* = 146]						
Low (< 44 U/L)	1 (0.7%)	259	259	0.899^a^	0.899	−0.005, 0.954
Normal (44–147 U/L)	72 (51.4%)	145 ± 121	117 (80–178)			
High (> 147 U/L)	67(47.9%)	137 ± 90	119 (75–189)			
TBIL [*N* = 142]						
Normal (0.1–1.2 mg/dL)	30 (22.1%)	182 ± 119	150 (100–199)	0.096	0.148	−0.28, 0.001
High (> 1.2 mg/dL)	111 (77.9%)	132 ± 103	115 (64–170)			
DBIL [*N* = 142]						
Normal (≤ 0.3 mg/dL)	16 (11.8%)	184 ± 146	150 (100–198)	0.123	0.148	−0.12, 0.148
High (> 0.3 mg/dL)	120 (88.2%)	138 ± 101	118 (75–181)			

Abbreviation: IQR, interquartile range; Sd, standard deviation.

^a^Only one patient in the “Low” category was excluded from this test.

^∗^P value is from ordinal regression with age and gender as covariates.

^∗∗^Benjamini–Hochberg procedure was used to adjust P values in order to control the False Discovery Rate (FDR).

^∗∗∗^Spearman′s rank correlation rho and p value.

Patients were also categorized based on the normal range for AST. The level of AST was found to be elevated in third of patients, whereas it was normal in the rest of the patients. None of the patients had a value lower than normal range as shown in Table 5.

FABP4 values in patients with high AST increased relative to the corresponding values in patients with normal AST as indicated in Table 4a. The median FABP4 level was higher in patients with elevated AST, in comparison to the median FABP4 in patients with normal AST. The difference in FABP4 between the normal and elevated AST groups was not significant.

Subdividing patients based on the normal range from more than 1 to 2 for aspartate aminotransferase to alanine aminotransferase (AST/ALT) ratio resulted in three groups as shown in Table 5.

Mean FABP4 values decreased with increasing AST/ALT ratio. The mean FABP4 in patients with AST/ALT lower than 1 was higher in patients with normal AST/ALT, mean FABP4, and in those with AST/ALT higher than 2 (Table 5). The differences in FABP4 levels between the categories of AST/ALT ratio were significant. Moreover, the correlation between AST/ALT ratio and FABP4 was negative and significant (*r* *h* *o* = −0.26, *p* = 0.001, adj. *p* = 0.003).

One patient had low ALP. The rest of patients were almost equally divided between either a normal ALP level or a high level as shown in Table 4a. Mean FABP4 was lower in high ALP group than in the normal ALP, but this difference was not significant as Table 5 indicated.

High levels of TBIL were found in the majority of patients, but the rest of the patients had normal levels as in Table 5. Mean FABP4 in the high TBILgroup was lower than the mean FABP4 in the normal group. The difference in FABP4 levels between normal and high TBIL is shown in Table 5. The correlation between TBIL and FABP4 was significant (*r* *h* *o* = −0.28, *p* = 0.001, adj. *p* = 0.003).

Results for DBIL were similar to those for TBIL. In this case too, the majority of patients had elevated DBIL, whereas the rest of the patients had normal DBIL levels as in Table 5. The same pattern of FABP4 levels in case of TBIL was also demonstrated with DBIL results. In other words, patients with high DBIL levels more than 0.3 mg/dL had lower mean FABP4 than patients with normal DBIL levels as Table 5 indicated.

Linear regression analysis of the log‐transformed ALT/AST ratio was conducted to identify independent predictors of liver disease severity (Table 6). Among the evaluated variables, only ferritin and FABP4 demonstrated significant associations with the ALT/AST ratio (*p* < 0.001 and *p* = 0.012, respectively). A 1000‐unit increase in ferritin corresponded to a 1.08‐fold higher ALT/AST ratio, indicating an approximately 8% increase. Similarly, each unit increase in FABP4 was associated with a 0.1% (0.02%–0.2%) rise in the ALT/AST ratio.

**Table 6:** Linear regression analysis of liver function.

Predictors	Outcome: log(ALT/AST) *n* = 132 *R* ^2^/*R* ^2^ adjusted 0.271/0.242		
	Estimates	95% CI	*P*
FABP4	1.001	1.0002–1.002	**0.012**
Ferritin	1.00008	1.00005–1.0001	**< 0.001**
Age	1.00	0.99–1.01	0.870
Gender [Male]	0.92	0.79–1.08	0.302
Number transfused units	1.10	0.97–1.24	0.144

*Note:* One ALT/AST outlier was removed from this analysis to improve the quality of the regression model.

### 3.6. Relationship Between Kidney Function and FABP4

Patients were subdivided into low, normal, and high groups based on the normal range of kidney function.

**Figure 2:**
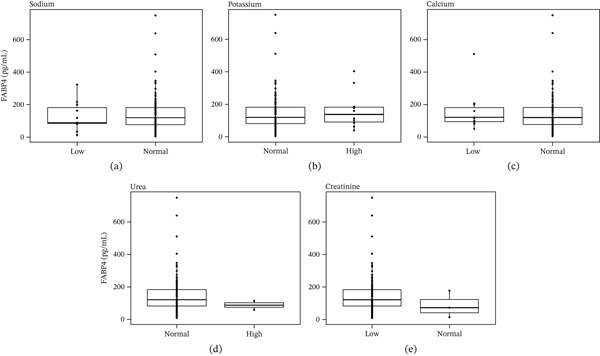
Association between serum FABP4 levels and biochemical parameters. Box‐and‐whisker plots show circulating FABP4 concentrations (pg/mL) across clinical categories of (a) sodium, (b) potassium, (c) calcium, (d) urea, and (e) creatinine. Boxes represent the interquartile range (IQR) with median values indicated by the central line, whiskers denote the minimum and maximum values, and individual dots represent outliers.

Eleven 7.6% of patients had low Na levels below 135 mmol/L, whereas the rest of patients had normal Na levels. Mean FABP4 was lower 127 ± 91 mmol/L in patients with low sodium than in patients with normal sodium 142 ± 107 mmol/L, but this difference was not significant Figure 2a.

On the other hand, 10 patients had elevated K > 5.2 mmol/L, whereas the rest of the patients had normal K levels. Except for one patient with a low potassium level. Patients with high potassium levels had a higher mean FABP4 165 ± 118 mmol/L than patients with normal potassium 140 ± 105 mmol/L. These differences in FABP4 were not significant of *p* value = 0.572 as Figure 2b indicated.

Moreover, Ca was found to be below the normal range < 2.1 mmol/L in 11 patients, whereas the rest of the patients were normal for Ca levels. The group of patients with low calcium had a higher FABP4 mean 162 ± 126 mmol/L than the group of patients with normal calcium 140 ± 107 mmol/L. Yet, this difference in FABP4 levels was not significant (*p* = 0.618) as in Figure 2c.

In the case of urea, the majority of the patients around 98.7% had normal levels, which leaves only two patients with elevated urea above 8.5 mmol/L. These two patients had lower mean FABP4 than the rest of the patients 85 ± 40 mmol/L versus 141 ± 106 mmol/L of *p* value = 0.398 as Figure 2d indicated.

Serum creatinine levels were below the normal range in the majority of patients (98%), with only three individuals exhibiting values within the normal limits. The low‐creatinine group demonstrated a mean FABP4 concentration of 142 ± 106 *μ*mol/L, compared with 84 ± 83 *μ*mol/L in patients with normal creatinine levels (*p* = 0.273), as shown in Figure 2e (Table 6). Linear regression analysis was subsequently performed to identify independent predictors of kidney function. FABP4 did not emerge as a significant predictor of creatinine in this model (*p* = 0.945) (Table 7).

**Table 7:** Categories of kidney function indicators in patients and the corresponding FABP4 levels.

Kidney function indicator	*n* (%)	FABP4		*P*	Rho, *p*
		*M* *e* *a* *n* ± *s* *d*	Median (IQR)		
Sodium [*N* = 145]					
Low (< 135 mmol/L)	11 (7.6%)	127 ± 91	88 (83–181)	0.695	−0.036, 0.665
Normal (135–145 mmol/L)	134 (92.4%)	142 ± 107	119 (76–181)		
Potassium [*N* = 145]					
Low (< 3.5 mmol/L)	1 (0.7%)	46.3	46.3	0.265 ^∗^	−0.012 0.885
Normal (3.5–5.2 mmol/L)	134 (92.4%)	140 ± 105	118 (79–181)		
High (> 5.2 mmol/L)	10 (6.9%)	165 ± 118	137 (89–183)		
Calcium [*N* = 137]					
Low (< 2.1 mmol/L)	11 (8.0%)	162 ± 126	120 (92–179)	0.771	−0.048 0.578
Normal (2.1–2.6 mmol/L)	126 (92.0%)	140 ± 107	118 (76–181)		
Urea [*N* = 147]					
Normal (2.1–8.5 mmol/L)	145 (98.6%)	141 ± 106	119 (79–181)	0.366	−0.02, 0.922
High (> 8.5 mmol/L)	2 (1.4%)	85 ± 40	85 (71–100)		
Creatinine [*N* = 145]					
Low (M: < 59, F: < 45 *μ*mol/L)	142 (98%)	142 ± 106	118 (80–182)	0.268	−0.014, 0.870
Normal (M: 59–104, F: 45–84 *μ*mol/L)	3 (2%)	84 ± 83	69 (40–122)		

## 4. The Diagnostic Ability of FABP4 for Beta‐Thalassemia

The ability of FABP4 to distinguish between healthy controls and beta‐thalassemia patients was investigated.

**Figure 3:**
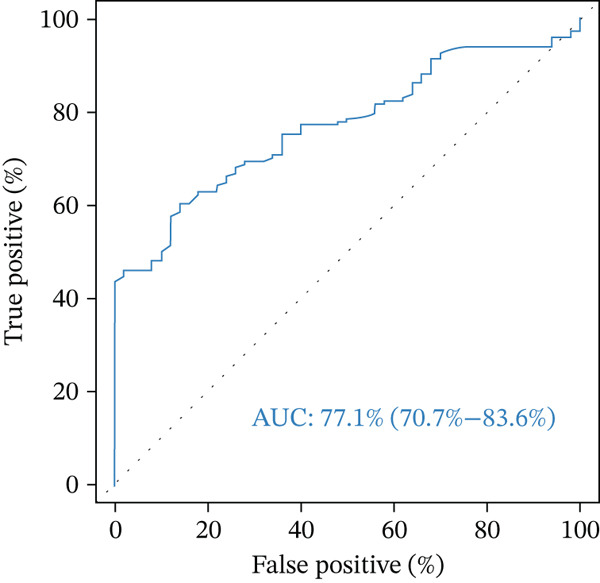
Receiver operating characteristic (ROC) curve of FABP4 for diagnosing *β*‐thalassemia. AUC: area under the curve. R Core Team (2022).

FABP4 demonstrated a moderate, but significant (*p* < 0.0001) ability to classify patients and healthy controls. The area under the receiver operating characteristic curve (AUC under the ROC curve) was 77.1% (70.7%–83.6%) as shown in Figure 3.

The best cut off value based on maximum Youden index was 102 pg/mL FABP4. This cutoff value resulted in 67% overall accuracy (correct classifications of patients and controls). On the other hand, a cutoff of 82 pg/mL had a slightly improved overall accuracy of 70%. The ability of these cutoff values to diagnose patients correctly was 60% and 72%, respectively, and their ability to recognize the absence of thalassemia was 86% and 64%, respectively.

## 5. Discussion

Thalassemia represents the most prevalent monogenic disorder globally. Early and regular blood transfusion therapy remains essential to minimize complications associated with severe anemia in affected patients. Iron overload in individuals with thalassemia major (TM) has increasing clinical attention due to strong association with elevated morbidity and mortality. Excessive iron deposition contributes to the generation of labile iron pools, enhancing the production of ROS and consequently promoting oxidative stress and progressive organ injury [30].

The present study included 154 participants, of whom the majority (56.5%) were male, representing various age groups. All patients demonstrated significantly higher serum FABP4 concentrations compared with the control group, consistent with the findings of Ezzat et al. [31]. A marked sex‐related difference in FABP4 levels was also observed, with female patients exhibiting higher concentration than males. This variation may be attributed to differences in adipose tissue distribution or to the modulatory effects of sex hormones on metabolic regulation, as reported in previous studies [32, 33].

Furthermore, serum FABP4 levels were notably elevated in participants aged 13–18 and 19–30 years, in agreement with the findings of González et al. [34]. The postpubertal period is commonly associated with reduce insulin sensitivity and increased fat mass, which may account for this observation, in addition, Tanaka et al. [35] reported elevated FABP4 levels among middle aged individuals, further supporting the age‐related trends observed in the current study but these differences were not significant.

Osteoporosis protrudes as the most prevalent comorbidity among Jordanian patients, aligning with other studies [36] who demonstrated the detrimental impact of osteoporosis on individuals with TM. Moreover, the current study revealed other health issues such as endocrine disorders, including diabetes, and hypothyroidism, among Jordanian patients. These findings are parallel with the reported results by another two groups [37] conducting that thalassemia patients commonly experience various chronic complications. Furthermore, cardiovascular and hepatobiliary diseases were found among the participants in those studies. Akiki et al. [38] associated those cardiac disorders that had significant morbidity and mortality implications for individuals with TM.

In hematological analysis of Jordanian patients with thalassemia, the levels of Hb, HCT, and RBCs were found to be below the normal reference range, consistent with the characteristic hematological features of thalassemia as reported by Munkongdee et al. [39] Conversely, NRBCs, WBCs, and PLTs were elevated in patients with TM. This increase may be attributed to splenectomy, which had been performed in 38.3% of the patients. Splenectomy is frequently associated with hematological alteration in individuals with thalassemia [40]. Moreover, splenomegaly in thalassemic patients induces hemolysis, extramedullary hematopoiesis, and iron overload as a consequence of recurrent blood transfusion, as documented by Taher et al. [41].

Serum ferritin levels in this study were markedly elevated, exceeding normal physiological limits. Elevated ferritin levels indicate iron overload, particularly among patients undergoing regular blood transfusion therapy. These findings are in line with previous research [42, 43] demonstrating that iron accumulation in various organs results from repeated transfusions and contributes to complications affecting the heart and liver. The ferritin levels of participants ranged from 253 to 12,269 ng/mL. However no significant correlation was observed between ferritin concentrations and FABP4 levels in this study. In contrast, Ezzat et al. [31] reported a positive correlation between ferritin and FABP4 among patients with ferritin levels exceeding 800 *μ*g/L, whereas fianza et al. [44] found weak inverse relationship between two markers.

ALT testing is widely utilized as a reliable indicator of hepatocellular integrity. In the present study, elevated ALT levels were observed in 35.7% of patients, reflecting possible hepatocellular injury of diverse etiologies. Typically, ALT assessment is performed concurrently with other hepatic biomarkers—such as AST, ALP, and bilirubin—to provide a comprehensive evaluation of liver function. AST elevation was detected in 37.8% of patients with thalassemia, among whom serum FABP4 levels were also increased. It is noteworthy that elevated AST is not invariably indicative of hepatic dysfunction, as transient increases may occur following strenuous physical activity [45]. Although the present study demonstrated a nonsignificant association between elevated ALT levels and serum FABP4 concentrations, ALT elevation remains more specific for hepatocellular injury secondary to iron overload, given the liver′s high susceptibility to iron‐induced oxidative damage.

AST elevation did not reach statistical significance, a finding consistent with the results reported by Rodríguez‐Calvo et al. [46]. However, the AST/ALT ratio demonstrated a significant association with serum FABP4 levels (*p* = 0.003). This observation concurs with the findings of Majhi et al. [47], who highlighted the diagnostic importance of the AST/ALT ratio in detecting hepatic dysfunction, as also supported by established clinical guidelines [48]. In the present study, 23.1% of patients exhibited an AST/ALT ratio greater than 2, whereas 29.4% of those with a ratio below 1 showed elevated FABP4 concentrations.

Chronic inflammation and hepatic injury secondary to iron overload may affect the AST/ALT ratio, indicating liver inflammation or fibrosis [49, 50]. Therefore, interpretation of liver enzyme results, including the AST/ALT ratio, should be integrated with other clinical and laboratory findings, as well as individual medical history and the specific characteristics of thalassemia. These results further support the potential of FABP4 as a biomarker reflecting chronic disease burden.

Bilirubin metabolism may also be impaired in thalassemia. Hyperbilirubinemia typically occurs due to extensive red cell destruction or hepatic dysfunction, as indicated by Fargo et al. [50]. Similarly, in this study, 77.9% of thalassemia patients presented with elevated TBIL levels, which corresponded with high FABP4 concentrations, showing significance (*p* = 0.003). This elevation likely results from the excessive destruction of abnormal erythrocytes and ineffective erythropoiesis, as described by Nienhuis and Nathan [51] and Bazvand et al. [52].

Renal function tests, including measurements of electrolytes (Na^+^ and K^+^), minerals (Ca^2+^), and waste products (urea and creatinine), are essential for assessing renal integrity and guiding patient management. In this study, serum FABP4 levels were lower among patients with hyponatremia than among those with normal sodium levels, whereas patients with hyperkalemia demonstrated higher FABP4 levels, though neither difference was statistically significant. Both hyponatremia and hyperkalemia are associated with chronic kidney disease (CKD), as reported by Kovesdy [53] and Yoon et al. [54].

A total of 98% of thalassemia patients exhibited low creatinine levels which could be mainly due to chronic liver disease, primely because the liver plays a central role in creatine synthesis, the precursor of creatinine Hartleb and Gutkowski [55], low serum creatinine with serum FABP4 mean levels of 140 pg/mL, consistent with the observations of Karim et al. [56] and Demosthenous et al. [57]. This finding contrasts with studies associating thalassemia with elevated creatinine levels, but it is supported by evidence linking reduced creatinine to higher transfusion intensity. Quinn et al. [58] also reported subclinical nephrotoxicity in thalassemia patients receiving iron chelation therapy, particularly deferasirox (Exjade), which was the most commonly used chelating agent in this study (77.9%).

FABP4 and ferritin emerged as independent predictors of liver disease severity, as reflected by ALT/AST ratio (*p* < 0.001), a finding consistent with the observations of Kowdley et al. [59].

## 6. Conclusion

A significant association was identified between serum FABP4 and bilirubin, and AST/ALT ratio, suggesting a possible relationship between serum FABP4 and hepatic injury as well as renal in thalassemia. These findings indicate a potential role of FABP4 in the pathophysiological processes underlying chronic organ involvement; however, this observation should be interpreted with caution. Further studies are required to confirm these associations and clarify the clinical significance of FABP4 in thalassemia.

## 7. Limitations

The current study forced to face several limitations, and these limitations were considered during the interpretation of the results. First, all samples were collected from a single clinical center, which may limit the ability to generalize the results to all thalassemia populations. Second, the patient enrolment process may introduce selection bias, as patients receiving consistent follow‐up care could differ from those not regularly monitored. Finally, the absence of longitudinal follow‐up data limits the evaluation of changes in FABP4 levels and their association with subsequent organ clinical complications.

**Nomenclature**
WHO: World Health Organization
FABPs: fatty acid‐binding proteins
KDa: kilo dalton
MOH: The Ministry of Health
Hb H: hemoglobin H
FDA: Food and Drug Administration
Dfo: Deferoxamine
DFX/DFRA: deferasirox
DFP: deferiprone
FABP4: fatty acid‐binding protein 4
aP2: adipocyte protein 2
NSCLC: nonsmall cell lung cancer
AKI: acute kidney injury
CKD: chronic kidney disease
ELISA: enzyme‐linked immunosorbent assay
Na: sodium
K: potassium
Ca: calcium
TBIL: total bilirubin
DBIL: direct bilirubin
ALP: alkaline phosphatase
AST: aspartate transaminase
ALT: alanine aminotransferase
WBC: white blood cell
RBC: red blood cell
Hb: hemoglobin
HCT: hematocrit
MCV: mean corpuscular volume
MCHC: mean corpuscular Hb concentration
MCH: mean corpuscular
PLTs: platelets
NRBC: number of nucleated RBCs
M: male
F: female
Ns: not significant
PBS: phosphate buffered saline
Plts: platelets
SD: standard deviation
CBC: complete blood count
Ng: nanogram
Pg: picogram
*μ*L: microliter
nm: nanometer
Streptavidin‐HRP: streptavidin‐horseradish peroxidase

## Funding

This study was supported by Al‐Ahliyya Amman University (10.13039/501100016299).

## Conflicts of Interest

The authors declare no conflicts of interest.

## Data Availability

The data that support the findings of this study are available on request from the corresponding author. The data are not publicly available due to privacy or ethical restrictions.
